# Elevation of Fasting Ghrelin in Healthy Human Subjects Consuming a High-Salt Diet: A Novel Mechanism of Obesity?

**DOI:** 10.3390/nu8060323

**Published:** 2016-05-26

**Authors:** Yong Zhang, Fenxia Li, Fu-Qiang Liu, Chao Chu, Yang Wang, Dan Wang, Tong-Shuai Guo, Jun-Kui Wang, Gong-Chang Guan, Ke-Yu Ren, Jian-Jun Mu

**Affiliations:** 1 Cardiovascular Department, Shaanxi Provincial People’s Hospital, Xi’an 710068, China; zhangyong971292@163.com (Y.Z.); cardiowang@163.com (J.-K.W.); ntxhx2005@stu.xjtu.edu.cn (G.-C.G.); 2 Cardiovascular Department, First Affiliated Hospital of Medical College, Xi’an Jiaotong University, Xi’an 710061, China; iaacd@163.com (C.C.); wangyangxxk@126.com (Y.W.); m15991631129@163.com (D.W.); 215guotongshuai@163.com (T.-S.G.); peizirong@yeah.net (K.-Y.R.); 3 Cardiovascular Department, Second Affiliated Hospital, Xi’an Medical University, Xi’an 710038, China; lfenxia@sina.com

**Keywords:** ghrelin, high salt, obesity, diet intervention

## Abstract

Overweight/obesity is a chronic disease that carries an increased risk of hypertension, diabetes mellitus, and premature death. Several epidemiological studies have demonstrated a clear relationship between salt intake and obesity, but the pathophysiologic mechanisms remain unknown. We hypothesized that ghrelin, which regulates appetite, food intake, and fat deposition, becomes elevated when one consumes a high-salt diet, contributing to the progression of obesity. We, therefore, investigated fasting ghrelin concentrations during a high-salt diet. Thirty-eight non-obese and normotensive subjects (aged 25 to 50 years) were selected from a rural community in Northern China. They were sequentially maintained on a normal diet for three days at baseline, a low-salt diet for seven days (3 g/day, NaCl), then a high-salt diet for seven days (18 g/day). The concentration of plasma ghrelin was measured using an immunoenzyme method (ELISA). High-salt intake significantly increased fasting ghrelin levels, which were higher during the high-salt diet (320.7 ± 30.6 pg/mL) than during the low-salt diet (172.9 ± 8.9 pg/mL). The comparison of ghrelin levels between the different salt diets was statistically-significantly different (*p* < 0.01). A positive correlation between 24-h urinary sodium excretion and fasting ghrelin levels was demonstrated. Our data indicate that a high-salt diet elevates fasting ghrelin in healthy human subjects, which may be a novel underlying mechanism of obesity.

## 1. Introduction

Obesity has serious health consequences and is, therefore, a public health concern worldwide, according to the World Health Organization. By 2014 obesity was more than double what it had been in 1980. Of the 1.9 billion overweight adults in 2014, 600 million were classified as obese. Obesity at one time was a problem only in wealthy countries, but today it is common in low- and middle-income countries as well, particularly in the cities [1]. Health dangers caused by obesity include cardiovascular disease, the leading cause of death worldwide in 2012, diabetes mellitus, osteoarthritis, other degenerative joint diseases, and some forms of cancer, in particular endometrial, breast, and colon cancer. Increasing evidence shows that in both humans and experimental animals, obesity, like hypertension, is related to high salt consumption [2,3,4,5,6,7,8,9,10,11]. The belief that elevated sodium intake is a cause of obesity appears to have its origins in the idea that salt intake may increase the desire to eat certain types of foods and beverages, thus contributing to weight gain [2,3,8]. However, the pathophysiologic mechanisms remain unresolved.

Ghrelin is a 28-amino-acid peptide with an *N*-octanoyl alteration at serine-3, produced mainly in the stomach, which has been identified as the endogenous ligand of the growth hormone (GH) secretagogue receptor (GHS-R) [12]. Ghrelin has various physiological functions, such as stimulating the release of growth hormone and of appetite, as well as fat accumulation. Administration of exogenous ghrelin reportedly enhances appetite and increases food intake through the activation of hypothalamic neuropeptide Y/agouti-related peptide neurons, expressing GHS-R type 1a. In addition, ghrelin controls glucose homeostasis by regulating insulin secretion and sensitivity in pancreatic beta cells. Elimination of ghrelin in obese diabetic *ob/ob* mice increases basal insulin concentrations, enhances glucose-stimulated insulin secretion, and improves peripheral insulin sensitivity [12,13,14,15].

Data are still scarce in the literature with regard to the link between salt intake and ghrelin. We hypothesized that ghrelin contributes to the progression of obesity during high-salt loading. In the present study, which included non-obese and normotensive subjects, we investigated whether salt intake influences fasting ghrelin levels.

## 2. Materials and Methods

### 2.1. Subjects

From a total of 50 screened, normotensive subjects with similar dietary customs from a rural community in Northern China, 38 were enrolled in this study, and 12 were excluded because of hypertension or obesity. A brief medical questionnaire was administered. Subjects with a previous history of hypertension, obesity, liver, or renal disease or with diabetes mellitus were excluded. Hypertension was defined as mean systolic blood pressure ≥140 mmHg and/or mean diastolic blood pressure ≥90 mmHg. Obesity was defined as BMI ≥ 28 kg/m^2^. All subjects were non-smokers. The institutional ethics committee of Xi’an Jiaotong University Medical School (Code: XJTU1AF2013LSL-056) approved the study protocol, and each subject gave written informed consent. All of the procedures were performed in accordance with institutional guidelines.

### 2.2. Protocol

The protocol consisted of a series of investigations, including a three-day baseline period in which a clinical history and physical examination (height, weight, and blood pressure (BP)) were obtained and subjects consumed a normal-salt diet, followed by seven days of a low-salt diet (51.3 mmol or 3 g of NaCl per day), then 7 days of a high-salt diet (307.7 mmol or 18 g of NaCl per day) [16]. During the baseline investigational period, each subject was given detailed dietary instructions to avoid table salt, cooking salt, high-sodium foods, and food rich in nitrite/nitrate for the subsequent 14 days. All meals were prepared in research kitchens and consumed onsite. Dietary total energy intake was supplied according to their baseline energy intake assessed by a brief food frequency questionnaire. Food consumption of study participants was carefully recorded at each meal by study staff members.

### 2.3. Biochemical Analyses

Blood glucose was measured using the glucose oxidase method. Serum lipids (total cholesterol, triglycerides, HDL-C) were measured. Blood samples for measurement of fasting plasma ghrelin concentrations were drawn with EDTA-aprotinin tubes and immediately placed on ice. All tubes were centrifuged at 4 °C for collection of plasma and stored at −80 °C until the time of analysis. Ghrelin was determined using a validated sandwich ELISA using a ghrelin-specific antibody (Wuhan USCN Science and Technology Co., Ltd., Wuhan, China). Five plasma samples were used to evaluate intra- and interassay coefficients of variation, which, for ghrelin, ranged from 2.1%–4.3% (mean, 3.2) to 6.4%–9.2% (mean, 7.8), respectively.

### 2.4. 24-h Urinary Sodium and Potassium Determination

Twenty-four-hour urine samples were collected at baseline and on day seven of each intervention period. Twenty-four hour urine collection was obtained with the first voided urine upon waking on the day of collection being discarded and participants then collecting all voided urine up to, and including, the first void the following morning. Participants were instructed to keep collected samples inside cooler bags provided and stored in a cool, dark place until completion when a research assistant was contacted to collect the sample. The times at the beginning and the end of urine collection were recorded. The samples were kept frozen at −40 °C until analysis. Urinary concentrations of sodium were determined using ion-selective electrodes (Hitachi, Ltd., Tokyo, Japan). The 24-h urinary excretion of sodium and potassium was calculated by multiplying the concentration of sodium and potassium, respectively, by the 24-h total urine volume.

### 2.5. Statistical Analysis

The data are shown as mean ± SD. Differences between biochemical markers obtained at low- and high-salt intakes were calculated by analysis of variance with the repeated measures design. Age, sex, and body mass index (BMI) were adjusted using multivariable analysis. All calculations were performed with SPSS for Windows 16.0 software (SPSS, Inc., Chicago, IL, USA). Probability was assessed using a two-tailed *p* value of < 0.05 to describe statistical significance.

## 3. Result

### 3.1. Profiles of Study Subjects

All enrolled subjects completed this interventional study. Their average age was 50.9 ± 1.3 years and systolic BP and diastolic BP were 110.7 ± 2.2 and 72.6 ± 1.3 mmHg, respectively (Table 1). The 24-h urinary sodium excretion averaged 173.8 mmol/day (median: 131.5), which approximates an intake of 8 g of salt/day. Daily urinary potassium excretion averaged 48 ± 0.9 mmol/day (median: 44 mmol/day).

### 3.2. Effects of Salt Intake on BP and 24-h Urinary Sodium Excretion

Table 2 shows BP responses to the low-salt and high-salt dietary interventions. BP significantly increased with the change from the low-salt to high-salt intervention (*p* < 0.05). The 24-h sodium excretions in the urine were calculated at the end of the intervention period to ensure compliance with the study protocol. As shown in Table 2, urinary sodium excretion significantly decreased with the change from baseline to the low-salt diet, but increased with the change from the low-salt to the high-salt diet (all *p* < 0.05). These results confirmed the subjects’ compliance with the dietary intervention protocol.

### 3.3. The Effect of High-Salt Intake on Fasting Ghrelin Levels

**Figure 1:**
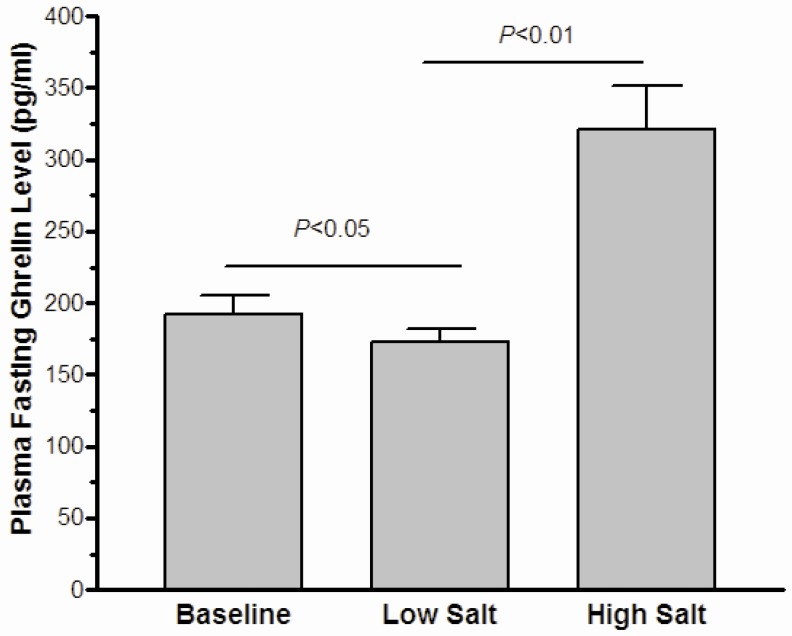
The effect of low-salt and high-salt intake on fasting ghrelin in all subjects.

**Figure 2:**
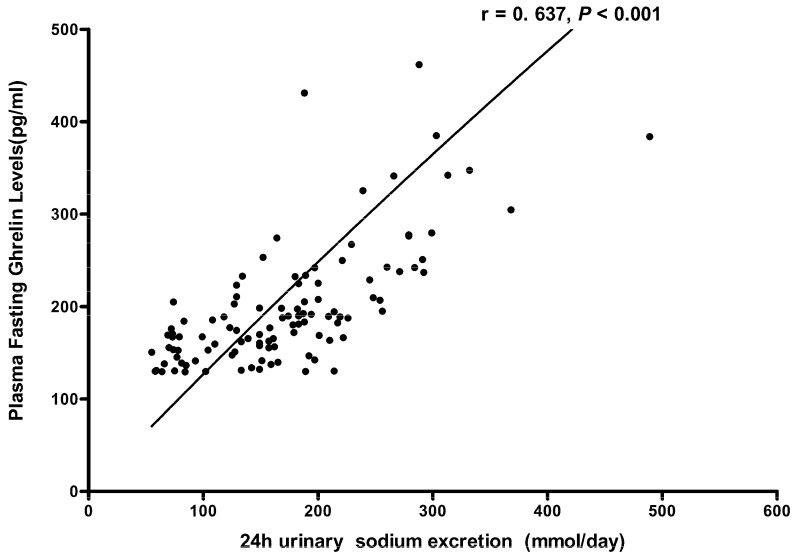
The correlation between plasma ghrelin levels and 24 h urinary sodium excretions in all subjects on baseline, a low-salt diet and on a high-salt diet.

As shown in Figure 1, the statistical difference was slight in the fasting ghrelin levels between the baseline and the low-salt dietary intervention (192.4 ± 12.6 pg/mL *vs*. 172.9 ± 8.9 pg/mL, *p* = 0.037). Moreover, high-salt intake clearly enhanced plasma ghrelin levels (320.7 ± 30.6 pg/mL *vs*. 172.9 ± 8.9 pg/mL, *p* < 0.01). Further analyses (Figure 2) showed that the fasting ghrelin concentration correlated with the 24-h urinary sodium excretion during among baseline, low-salt, and high-salt dietary intervention periods (*r* = 0.637, *p* < 0.001).

## 4. Discussion

The initial finding of the present study is that high-salt intake is associated with fasting ghrelin elevation in non-obese and normotensive subjects. In addition, a positive correlation between 24-h urinary sodium excretion and fasting ghrelin levels was demonstrated in these Chinese subjects.

The well-established role of ghrelin as an important regulator of appetite and fat accumulation may be attributed to the pathogenesis of obesity. Chronic ghrelin administration increases body weight via diverse, concerted actions on food intake, energy expenditure, and fuel utilization [17,18,19,20,21,22,23]. On the contrary, congenital ablation of the ghrelin or ghrelin-receptor gene causes resistance to diet-induced obesity [24,25,26], and pharmacologic ghrelin blockade reduces food intake and body weight [21,27,28,29,30]. There may be a relationship between the genomic variation in the ghrelin gene and obesity [21,22,23,24,25,26,27,28,29,30,31,32,33]. In particular, the Leu72Met polymorphism in ghrelin was found to be associated with the early onset of obesity [31]. Ghrelin is produced primarily in the stomach, circulates in the blood, and serves as a peripheral signal, informing the arcuate nucleus of the central nervous system (via the vagus nerve) to stimulate hunger. Moreover, ghrelin also could be involved glucose metabolism through suppressing insulin secretion, which insulin plays a crucial role in obesity. It has been well documented that systemic administration of ghrelin or endogenous ghrelin could restrict glucose-induced insulin release and deteriorate glucose tolerance *in vivo* and *in vivo* [34,35,36], which leads to insulin resistance, indirectly take part in obesity. Therefore, ghrelin may possibly be a useful target against obesity.

Ghrelin may be the key to revealing the mechanisms of salt-induced obesity. Several epidemiological studies have demonstrated a clear relationship between salt intake and obesity [2,6,7], and interventional studies also show that a high-salt diet might induce subcutaneous and visceral adipose tissue [4,11]. A hypothesis has been proposed to explain the relationship between sodium intake and obesity: salty foods might be considered addictive substances that stimulate opioid receptors in the brain and the pleasure center, so that the consumption of salty foods every day produces an addiction to these foods, producing an increase in food consumption (tolerance to opiates), increased caloric intake, overweight, sedentary lifestyle, obesity, and related diseases [37,38]. In our study, we found that the high-salt diet elevated fasting ghrelin. Recently, Cai *et al*. reported that ghrelin is co-expressed with ENaC subunits in taste-receptor cells in fungiform papillae on the tongue and that Ghrelin/GHS-R/mice possess a significantly reduced salty taste sensitivity compared to wild-type mice [39]. Therefore, we conclude the salt-induced ghrelin increase may be a novel mechanism of obesity via informing the arcuate nucleus of the central nervous system to stimulate hunger.

Our study has several important strengths. First of all, the subjects were recruited from a rural community and were similar with respect to lifestyle and environmental risk factors, including diet and physical activity. Thus, confounding due to these exposures should be minimized. Moreover, participants with body mass index (BMI) more than 28 kg/m^2^ was excluded, and adjustment for BMI was conducted; therefore, confounding effects of BMI probably had no influence on salt intake and ghrelin. Participation in the dietary interventions was high, and compliance with the study interventions, as assessed by urinary excretion of sodium during each intervention period, was excellent.

The present study has some limitations that should be addressed. Because all of the subjects were recruited from the Chinese population, whether or not our observation could be generalized to other racial populations is unknown. Further studies are required to validate our findings in a larger and more diverse sample and to elucidate the mechanisms by which salt loading affects plasma ghrelin. Meanwhile, insulin resistance could be involved in salt-induced obesity, and further studies will be needed to reveal the relationship between ghrelin and insulin during high-salt diet.

## 5. Conclusions

In conclusion, our human intervention study found that salt loading could increase circulating ghrelin production in normotensive Asian subjects. Our findings indicate that the elevation of ghrelin might be the underlying mechanism of salt-induced obesity, which sheds some new light on prevention and a possible therapeutic target for obesity in the future.

## Figures and Tables

**Table 1:** Baseline demographic and clinical characteristics.

Parameter	Values
Mean age (year)	50.6 ± 2.1
Sex (male/female)	21/17
BMI (kg/m^2^)	22.8 ± 0.4
Systolic BP (mmHg)	110.6 ± 5.8
Diastolic BP (mmHg)	72.1 ± 2.7
Glucose, mmol/L	3.91 ± 0.11
Total cholesterol, mmol/L	4.18 ± 0.14
Triglycerides, mmol/L	1.32 ± 0.11
LDL-cholesterol, mmol/L	2.35 ± 0.11
HDL-cholesterol, mmol/L	1.21 ± 0.04

**Table 2:** BP Levels (mmHg) and 24-h Urinary Sodium (mmol/day) at Baseline and During Dietary Interventions.

	SBP	DBP	24 h Urinary Na^+^ (mmol/Day)
Baseline	110.6 ± 5.8	72.1 ± 2.7	175.8 ± 11.1
Low-salt diet	108.7 ± 2.8	73.5 ± 2.0	98.8 ± 9.3
High-salt diet	116.4 ± 5.8 *	77.3 ± 4.2 *	268 ± 10.2 *

* *p* < 0.05 *versus* low-salt diet.
